# The Potential for a Released Autosomal X-Shredder Becoming a Driving-Y Chromosome and Invasively Suppressing Wild Populations of Malaria Mosquitoes

**DOI:** 10.3389/fbioe.2021.752253

**Published:** 2021-12-03

**Authors:** Yehonatan Alcalay, Silke Fuchs, Roberto Galizi, Federica Bernardini, Roya Elaine Haghighat-Khah, Douglas B. Rusch, Jeffrey R. Adrion, Matthew W. Hahn, Pablo Tortosa, Rachel Rotenberry, Philippos Aris Papathanos

**Affiliations:** ^1^ Department of Entomology, Robert H. Smith Faculty of Agriculture, Food and Environment, Hebrew University of Jerusalem, Rehovot, Israel; ^2^ Department of Life Sciences, Imperial College London, London, United Kingdom; ^3^ Centre for Applied Entomology and Parasitology, School of Life Sciences, Keele University, Keele, United Kingdom; ^4^ Center for Genomics and Bioinformatics, Indiana University, Bloomington, IN, United States; ^5^ Institute of Ecology and Evolution, University of Oregon, Eugene, OR, United States; ^6^ Department of Biology, Indiana University, Bloomington, IN, United States; ^7^ Department of Computer Science, Indiana University, Bloomington, IN, United States; ^8^ Unité Mixte de Recherche Processus Infectieux en Milieu Insulaire Tropical (UMR PIMIT), Université de La Réunion, INSERM 1187, CNRS 9192, IRD 249, Plateforme de Recherche CYROI, Saint Denis, France; ^9^ Centers for Disease Control and Prevention, Atlanta, GA, United States

**Keywords:** gene drive, malaria, sex-ratio distortion, genetic control, risk assessment

## Abstract

Sex-ratio distorters based on X-chromosome shredding are more efficient than sterile male releases for population suppression. X-shredding is a form of sex distortion that skews spermatogenesis of XY males towards the preferential transmission of Y-bearing gametes, resulting in a higher fraction of sons than daughters. Strains harboring X-shredders on autosomes were first developed in the malaria mosquito *Anopheles gambiae*, resulting in strong sex-ratio distortion. Since autosomal X-shredders are transmitted in a Mendelian fashion and can be selected against, their frequency in the population declines once releases are halted. However, unintended transfer of X-shredders to the Y-chromosome could produce an invasive meiotic drive element, that benefits from its biased transmission to the predominant male-biased offspring and its effective shielding from female negative selection. Indeed, linkage to the Y-chromosome of an active X-shredder instigated the development of the nuclease-based X-shredding system. Here, we analyze mechanisms whereby an autosomal X-shredder could become unintentionally Y-linked after release by evaluating the stability of an established X-shredder strain that is being considered for release, exploring its potential for remobilization in laboratory and wild-type genomes of *An. gambiae* and provide data regarding expression on the mosquito Y-chromosome. Our data suggest that an invasive X-shredder resulting from a post-release movement of such autosomal transgenes onto the Y-chromosome is unlikely.

## Introduction

Mosquito species of the *Anopheles gambiae* complex are the main vectors of human malaria and pose an enormous burden on global health and economies (World malaria report, 2020). The progressive spread of insecticide resistant mosquitoes (Hyde, 2005; Sinha et al., 2014) has prompted the development of new methods to control these mosquitoes (Windbichler et al., 2008; Kyrou et al., 2018; Carballar-Lejarazú et al., 2020). One of the most promising is genetic control, which is based on the release of laboratory-modified insects into the environment. Released individuals mate with wild insects and transmit control traits that can suppress or modify the targeted population (Hamilton, 1967; Curtis, 1968). Among these, the most commonly used approach to genetically control insects has been the mass release of sterile males—the so-called Sterile Insect Technique (SIT) (Knipling, 1959; Wyss, 2000). When wild monandrous females mate with released sterile males, their eggs are fertilized by sperm carrying mutations that abort embryo development. If sufficient numbers of sterile males are released over a long enough period, the wild population can be effectively suppressed or even eradicated. However, the economic costs of an SIT program that aims for mosquito suppression in very large areas and the need to maintain sterile releases indefinitely, have restricted the implementation of this method to date [but see; (Zheng et al., 2019, Hendrichs et al., 2021; Balatsos et al., 2021)].

One way to improve the efficiency of such approaches is through the release of fertile males that are daughterless. Since male mosquitoes do not contribute to disease transmission, releasing males that have viable and fertile sons can help to temporarily maintain the frequency of the allele or transgene in the population, which in turn helps to reduce the abundance of females. Two strategies based on such fertile males have been developed in mosquitoes thus far: fs-RIDL (for female-specific Release of Insects carrying Dominant Lethals) and sex ratio distorters based on X-chromosome shredding (Thomas et al., 2000; Burt, 2003; Phuc et al., 2007; Windbichler et al., 2007; Galizi et al., 2014). fs-RIDL is based on a construct that is lethal to females that inherit it, so that daughters of released transgenic males born in the field and inheriting the transgene die before maturing or are unable to fly (flightless), but sons survive and pass the transgene to their offspring (Thomas et al., 2000; Phuc et al., 2007). Sex-ratio distortion based on X-chromosome shredding instead, relies on the expression of a sequence-specific endonuclease during male spermatogenesis that recognizes and cleaves sequences that are both specific and abundant on the X-chromosome (Windbichler et al., 2007; Galizi et al., 2014). As a result, X-chromosome-bearing gametes are eliminated from the viable sperm population, thus biasing offspring sex-ratios towards males (Burt, 2003; Deredec et al., 2008; Papathanos and Windbichler, 2018; Haghighat-Khah et al., 2020). Mathematical models predict that both approaches are more efficient than SIT in terms of the number of modified males that need to be released to achieve a similar level of population suppression (Schliekelman et al., 2005; Burt and Deredec, 2018). Despite being more efficient, both fs-RIDL and autosomal X-shredders (where the transgene is located on an autosome) are self-limiting. The transgenic constructs underlying the phenotype will therefore not spread in the population, because they are inherited in a Mendelian fashion and do not provide any fitness advantage over the wild type. This is different for self-sustaining approaches such as those incorporating gene drive constructs (Alphey, 2014; Hammond and Galizi, 2017). The fact that in X-shredding, the X-chromosome-bearing gametes are eliminated pre-zygotically can be used for self-sustaining genetic control applications, in the form of Y-chromosome drive as originally proposed by Hamilton (Hamilton, 1967). This could be done by linking a functional X-shredder to the Y-chromosome, in which case both the Y-chromosome and the X-shredder gain a transmission advantage through preferential inheritance of male-forming gametes (Deredec et al., 2011).

**FIGURE 1:**
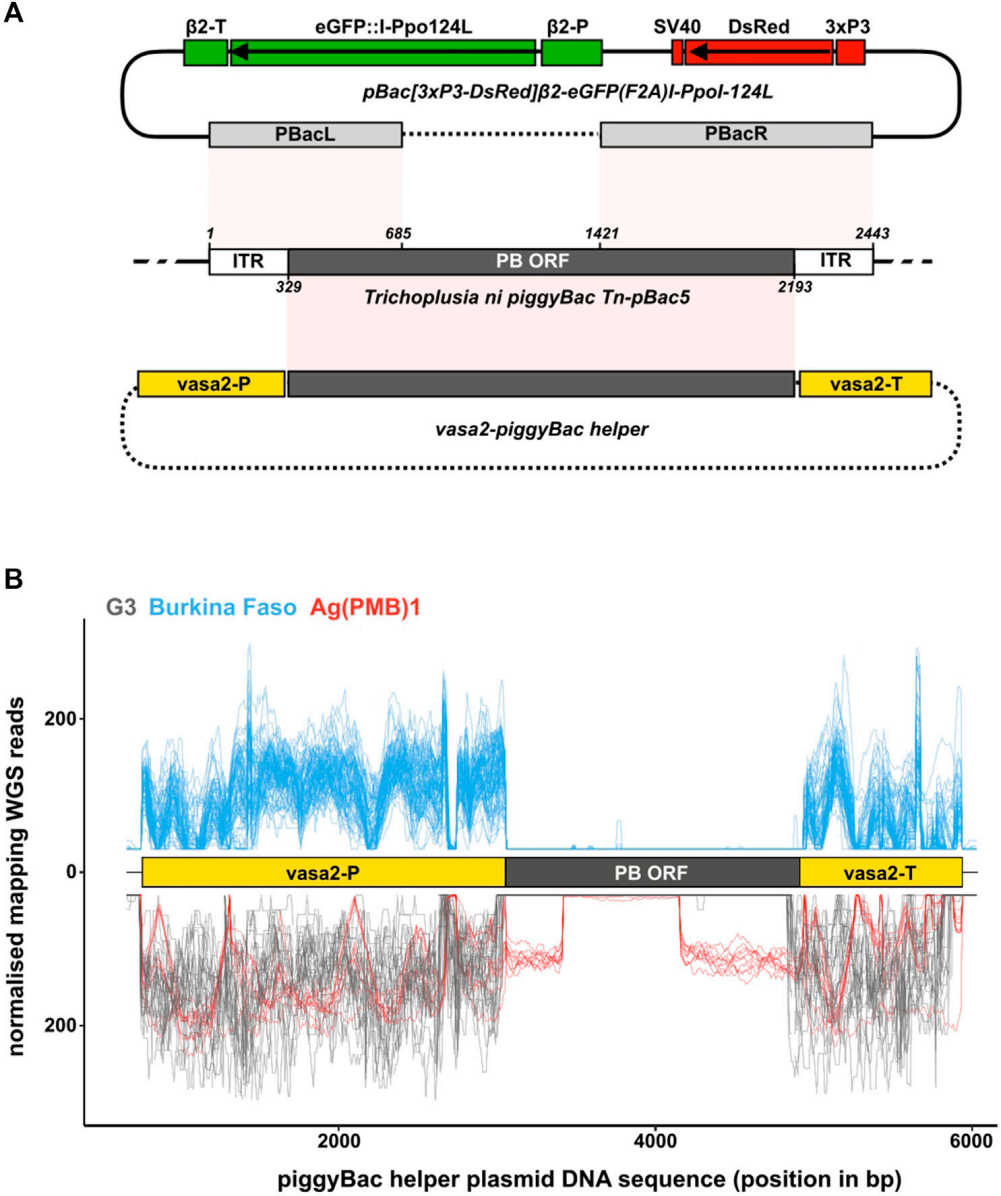
piggyBac transposase components in laboratory and wild type individuals. **(A)** Schematic of a wild type piggyBac (PB) transposon from *Trichoplusia ni* (middle; NCBI accession DQ236240.1), the Ag(PMB)1 transformation construct [(Galizi et al., 2014); top] and the PB helper plasmid [(Volohonsky et al., 2015); bottom]. Shown are the regions of the endogenous PB locus present in the two microinjected plasmids highlighting how both the transformation and helper plasmid lack the complete machinery required for transposon mobility. pBacL **(left)** and pBacR **(right)** arms present in the pBac[3xP3-DsRed]β2-eGFP:I-*Ppo*I-124L transformation construct contain the entire flanking inverted terminal repeats (ITRs) and partial regions of the PB open reading frame (ORF). The helper PB plasmid containing the complete PB ORF driven from the *vasa*2 regulatory regions lacks the flanking ITRs. Sequences of the transformation construct that are integrated in the genome, and those present only transiently in injected individuals are shown in solid lines and dashed lines, respectively. **(B)** Mapping of whole genome sequencing reads from the G3 and Ag(PMB)1 controls (**bottom** in negative y-axis; grey and red) and the 81 wild type individuals collected in Burkina Faso villages (**top**; blue). The position of the *An. gambiae vasa* regulatory regions (yellow boxes) and the PB transposase ORF (black box) is also shown. Reads are normalized by scaling counts to the number of reads in the most abundant sample.

A X-shredding sex-distorter was first developed in *An. gambiae* by Galizi et al. (2014). They used variants of the I-*Ppo*I endonuclease that cut a specific DNA target sequence within the 28S ribosomal DNA locus, which in *An. gambiae* is located exclusively on the X chromosome in approximately 200–400 copies (Collins et al., 1989). These I-*Ppo*I variants were fused to eGFP and driven by the *An. gambiae beta-2 tubulin* regulatory regions, which become active in primary spermatocytes entering male meiosis (Catteruccia et al., 2005). The resulting transformation constructs also included the *DsRed* transformation marker driven by the neuron-specific *3xP3* promoter, and the entire cassette was flanked by piggyBac-specific left and right arms containing the inverted terminal repeat sequences (ITRs) (Figure 1A). Of all the transgenic strains examined, ^gfp^124L-2, since renamed by the Target Malaria Research consortium as Ag(PMB)1 (for *An. gambiae* Paternal Male Bias strain 1) expressing the I-*Ppo*I structural variant W124L, showed high sex ratio distortion among progeny of transgenic males (approximately 95% males), without significantly impairing male fertility and fitness and is thus being currently evaluated for field testing by the Consortium (Galizi et al., 2014). Inverse PCRs produced as part of that study showed an autosomal location of the transgene, from where the sex-distortion phenotype was stably inherited over consecutive generations. In large cage experiments, weekly inoculative releases of transgenic Ag(PMB)1 males led to a reduction both in the egg productivity of the population and the frequency of females over successive generations consistent with model predictions (Facchinelli et al., 2019).

Since Ag(PMB)1 males are fertile, their release should result in viable offspring in the field, unlike sterile males. This would provide invaluable information about how transgenic, laboratory-reared males of *An. gambiae* disperse spatially once released and can be achieved even from small-scale releases aimed at capacity-building and methodology development. Unlike fs-RIDL strains that were directly developed for deployment, the lack of a conditional expression system or some other design to control activity of the X-shredder makes this strain unsuitable for large-scale programs aiming directly for population suppression, since rearing at large numbers is logistically difficult because this strain can only be maintained through females to avoid strain loss. Moreover, the Ag(PMB)1 X-shredder was not designed for gene drive in its current form - for example it is not able to home into targeted sequences. It also does not display any fitness advantage over wild type mosquitoes. Consistent with this, models predict that the Ag(PMB)1 transgene would disappear over time when releases are discontinued (Burt and Deredec, 2018) and recent large cage experiments confirmed the loss of the transgene from the population over time (Pollegioni et al., 2020).

One feature that is unique to an autosomal X-shredder compared to other self-limiting strategies, is the possibility that it could move to the Y-chromosome after release, potentially becoming self-sustaining in the form of a Y-chromosome drive as first coined by Hamilton (1967). If active, a Y-chromosome linked X-shredder could directly benefit from the increased transmission of the Y-chromosome due to preferential inheritance of male-forming gametes, thus increasing in frequency, persisting longer and dispersing further than initially planned. The sequential events required for such a driving Y to occur can be mapped to “pathways to harm” using a problem formulation approach adopted widely in environmental risk assessments (Supplementary Figures S1, S2). Three requirements must be fulfilled for a driving Y to occur: 1) the autosomal X-shredder must first move from its original autosomal position and become physically linked to the Y-chromosome; 2) the X-shredder would need to be expressed from its new position on the Y-chromosome during late spermatogenesis in a spatiotemporal manner that is similar to its original expression from the autosome; and 3) it should impart no significant cost to male fertility or male viability as a result of its new Y-chromosome-linkage (Supplementary Figures S1, S2).

With regard to requirement 1), excluding any DNA repair mechanisms as this would require unlikely pairing of the autosome and the Y chromosome, there are two possible mechanisms that could result in an autosomal transgene moving to the Y-chromosome: 1) a transposase-mediated transposition to the Y-chromosome of the piggyBac (PB) transposable element that was used to create the transgenic strain, or 2) a recombination-mediated reciprocal translocation resulting in large chromosomal rearrangements between the autosome and the Y-chromosome. Of the two mechanisms, translocation is the less likely route, because translocations between autosomal segments and the Y-chromosome occur very rarely in nature (see Discussion). On the other hand, transposition from the autosome to the Y through the remobilization of the PB transposon could be possible, if the X-shredder transgene co-occurs in a genome containing an active PB transposase.

In this paper, we addressed the possibility of transgene remobilization by examining mosquito genomes for evidence of the PB transposase which recognize the inverted terminal repeats of the transgene and remobilize it. With regard to requirement 2), expression from the Y–we generated two independent transgenic strains containing the eGFP:I-*Ppo*I-124L X-shredder construct on the Y-chromosome and evaluated the level of expression and sex-ratio distortion. Finally, we discuss implications for male fertility depending on the route of movement to the Y-chromosome.

## Results

### Evaluating the Remobilization Potential of the Autosomal Ag(PMB)1 X-Shredder Transgene

The Ag(PMB)1 strain was generated by Galizi et al. (2014) by micro-injecting *An. gambiae* G3 embryos with a mixture of the transformation plasmid [pBac(3xP3-DsRed)β2-eGFP:I-*Ppo*I-124L] and a helper plasmid, containing the piggyBac (PB) transposase expressed from the *vasa* regulatory regions (Volohonsky et al., 2015), which direct expression in germline tissues (Figure 1A) (Papathanos et al., 2009). By providing the PB transposase in *trans* from a transiently co-injected helper plasmid, the transformation construct itself became immobilized once it integrated in the genome. This is because, unlike the complete PB transposable element, the transgene lacks the complete transposase enzyme that is required for remobilization. Therefore, integrated PB transgenic constructs can only be remobilized in mosquito transgenic strains, if a PB transposase source is available (O'Brochta et al., 2011).

To assess the stability of the Ag(PMB)1 transgene, we first evaluated whether the original insertion site, as described in Galizi et al. (2014), has remained stable in the approximately 100 generations since its initial generation in laboratory populations that are typically maintained by crossing transgenic females (approximately 200 per generation) to wild type males. We designed PCR primers that span the PB transgene and genomic boundary (flanking regions) as originally reported (Galizi et al., 2014), and repeated the PCR using genomic DNA from 162 heterozygous transgenic individuals, that were generated by crossing transgenic females to wild type males. Transgene inheritance on the basis of DsRed fluorescence was scored twice during larval development and found in half of larval offspring, as expected for a single copy of the transgene in the genome. Of the 162 transgenic individuals tested, all contained the transgene in the expected location, as indicated by successful amplification from primers annealing in internal and flanking sequences (Supplementary Figures S3, S4). These results suggest that either the Ag(PMB)1 transgene has not remobilized in the strain, or, if new alleles have emerged, that these are not represented at detectable levels using our laboratory assays designed to test the transgene location. This indicates that PB transposase does not occur naturally in the genome of the laboratory colony. It also suggests that none of the other naturally occurring transposable elements present in this strain are able to remobilize the Ag(PMB)1 transgene, in the absence of the initially provided PB-helper source.

Given this issue of detection at scale, we next tested whether we could detect the gene encoding PB transposase in the genomes of the G3 or Ag(PMB)1 strains. This would exclude the possibility that PB transposase gene is present but is either non-functional, e.g., through mutations in its open reading frame, or suppressed by gene silencing by piRNAs (Senti and Brennecke, 2010; Halic and Moazed, 2009). To do this, we generated whole genome sequence (WGS) libraries from genomic DNA extracted from 10 individuals (five females, five males) of the Ag(PMB)1 strain and downloaded WGS libraries from 24 previously sequenced G3 individuals from the same insectary colony (PRJNA397539). We mapped the WGS data to the PB helper plasmid that was originally used to generate the Ag(PMB)1 transgenic strain, containing the PB transposase driven by the 5′ and 3′ regulatory regions of *An. gambiae vasa* gene. Mapping WGS reads against the helper plasmid ensured that the coding sequence evaluated is experimentally verified to catalyze excision of PB transgenes, instead of a different transposable element that may be related at the sequence level but is unable to excise PB transgenes. The helper plasmid included internal positive controls, in the form of regulatory sequences from the endogenous single-copy *vasa* gene and parts of the flanking PB left and right arms of the Ag(PMB)1 transgene (Figure 1A). We observed a high number of mapping WGS reads from G3 samples against the *vasa*-derived regulatory sequences on the helper plasmid, but no continuous mapping in the region corresponding to the PB transposase enzyme (Figure 1B). For the Ag(PMB)1 strain, genomic reads mapped to both the endogenous *vasa* regulatory sequences and to internal sequences of the PB ORF that correspond with the parts of PB left and right arms included in the transformation construct used to generate Ag(PMB)1, as expected (Figure 1A). No reads were detected on the PB coding sequence that is excluded in the transformation construct (Figures 1A,B). We then repeated the same analysis using single-mosquito WGS data from 81 field-caught individuals collected in Burkina Faso in 2012 (NCBI BioProject Accession PRJEB1670), which is considered for a potential release of Ag(PMB)1 mosquitoes by the Target Malaria Consortium (Supplementary Figure S5) (Scudellari, 2019). Similar to the results from the G3 samples, no reads mapped to the part of the helper plasmid encoding the PB transposase open reading frame with reads mapping exclusively to the regions of the endogenous *vasa* gene. Together, these results suggest that the PB transposase is unlikely to be in the local genetic background of populations into which an introgressed autosomal Ag(PMB)1 transgene may be released in the future.

### X-Shredder Expression From the Y-Chromosome During Spermatogenesis

The second requirement for the Ag(PMB)1 X-shredder to display gene drive and invasiveness, assuming the transgene has first moved to the Y-chromosome, is that it is expressed in a correct spatiotemporal manner and level from its new location. In the Ag(PMB)1 strain, X-shredding is achieved through the expression of the eGFP:I-*Ppo*I-124L transgene from the *An. gambiae beta2-tubulin* regulatory regions, which is highly active shortly before the first meiotic division in primary spermatocytes, and continues throughout the subsequent stages of spermatozoa differentiation (Michiels et al., 1993). In previous work, we have shown that transgenes driven from this promoter are strongly expressed when located on *An. gambiae* autosomes, but when they are inserted on the X-chromosome, expression is undetectable (Magnusson et al., 2012). This includes various X-chromosome-linked X-shredder variants, where no significant expression or sex bias was observed (Galizi et al., 2014). Similar observations of X-linked transgene transcriptional suppression around meiosis have been made in other species (Hoyle et al., 1995; Hense et al., 2007; Kemkemer et al., 2014). This phenomenon, called meiotic sex chromosome inactivation (MSCI), is thought to be one of the main driving forces leading to the observed paucity of sperm-specific genes on the X-chromosome, both in *An. gambiae* mosquitoes and in other species (Magnusson et al., 2011; Papa et al., 2017; Taxiarchi et al., 2019). By comparison, much less is known about transgene expression during spermatogenesis from the *An. gambiae* Y chromosome, which is estimated to be around 26 Mbp long, approximately 10% of the mosquito genome (Bernardini et al., 2017), and is composed nearly entirely of a few massively amplified, tandemly arrayed repeats and five known genes (Hall et al., 2016).

**FIGURE 2:**
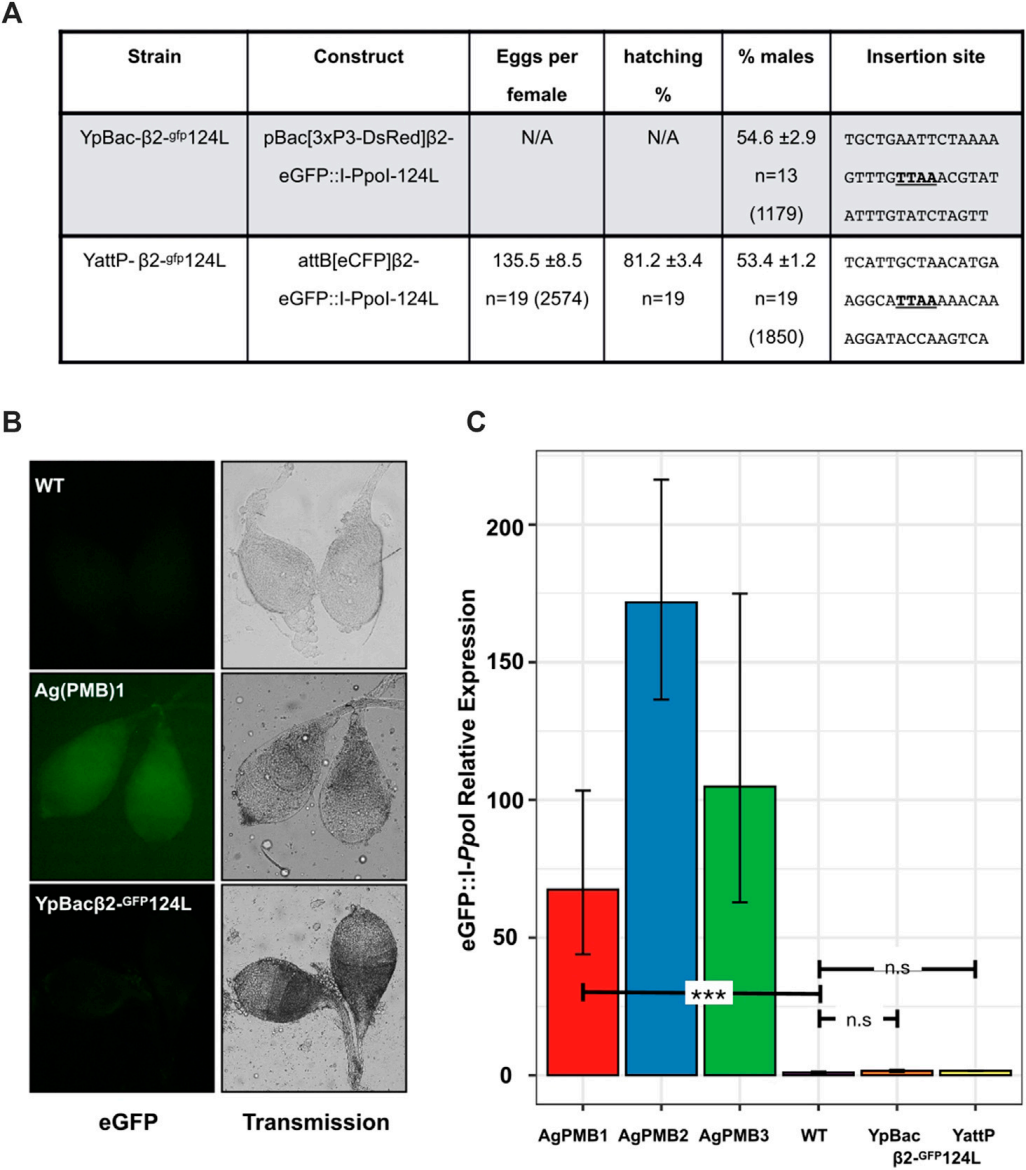
Transcriptional suppression of Y-linked X-shredder constructs abolishes sex ratio distortion. **(A)** Progeny analysis of males from the two Y linked X-shredder strains crossed to wild-type females. Shown is the average number of eggs laid per n females analyzed (±represents the standard error of the mean; SEM). Average percentage of larvae hatching from the eggs (±SEM), from *n* females analyzed. Average percentage of males in the progeny (±SEM) from *n* females. The total number of eggs or individuals counted in each experiment is given in parentheses. Sequences (20 bp each side) flanking the PB integration site (TTAA) of the transformation constructs are also shown. **(B)** eGFP fluorescence from dissected wild type (WT), Ag(PMB)1 and YpBac-β2-^gfp^124L testis. **(C)** Quantitative RT-PCR showing the relative expression of eGFP:I-PpoI variants in autosomal X-shredder strains [Ag(PMB)1-3] and Y-linked X-shredder strains. Expression levels were normalized to G3 wild-type (RQ = 1) which contains no I-*Ppo*I component. Expression of the X-shredder is undetectable in both Y-chromosome insertions compared to G3 wild-type (unpaired t-test *p* = 0.1669 for YpBac and *p* = 0.2509 for YattP). Expression levels from autosomal strains, Ag(PMB)1 (unpaired t-test *p* = 0.0078), Ag(PMB)2 (originally W124L-3) and Ag(PMB)3 (originally L111A-2) which led to sex ratio distortion are shown.

To test whether MSCI has a similarly inhibitory effect on transgene expression during spermatogenesis on the Y-chromosome as the X-chromosome, we generated two independent transgenic strains harboring the Ag(PMB)1 X-shredder, eGFP:I-*Ppo*I-124L, on the *An. gambiae* Y-chromosome. The first transgenic strain, called YpBac-β2-^gfp^124L, was generated by random PB integration. We sequenced the insertion site of the YpBac-β2-^gfp^124L transgene by inverse PCR on genomic data extracted from transgenic males and found that the construct had inserted within the highly-abundant Y-chromosome-specific transposable element *zanzibar* (Hall et al., 2016) (Figure 2A). The second transgenic strain, called YattP-β2-^gfp^124L, was obtained by secondary φC31 site-specific integration into an AttP docking site we previously inserted on the Y-chromosome (Bernardini et al., 2014). Similar to the YpBac-β2-^gfp^124L strain, the AttP site is located in a region of the Y-chromosome containing the *zanzibar* repeat, though it is not possible to estimate the distance between these two insertions given the lack of a continuous Y-chromosome genome assembly (Hall et al., 2016).

As would be expected from Y-linked insertions, transgenic offspring from males of both Y-linked strains and wild-type G3 females were exclusively males. Testes from transgenic males of both strains displayed no obviously detectable eGFP signal by fluorescence microscopy above background auto-fluorescence, which would be expected if the eGFP:I-*Ppo*I-124L X-shredder transgene was expressed (Figure 2B), and overall testes fluorescence was indistinguishable from testes of wild type males in all individuals tested over multiple generations. These strains have, and still are under observation for Y-expression. In total, hundreds of observations have been made over more than 40 generations since they were generated. Conversely, expression from the 3xP3-DsRed transformation markers in both strains was phenotypically indistinguishable from autosomal insertions, suggesting that Y-chromosome linkage does not interfere with somatic expression of transgenes, at least from these two positions (Bernardini et al., 2014). To quantify this observation, we next analyzed the levels of eGFP:I-*Ppo*I-124L transcription in the testes of the YattP-β2-^gfp^124L and YpBac-β2-^gfp^124L strains. As a control, we also evaluated expression in testes of two additional strains from the Galizi et al. (2014) study [^gfp^124L-3 and ^gfp^111A-2, called here Ag(PMB)2 and Ag(PMB)3, respectively] and wild-type males. Results from the quantitative RT–PCRs show no significant levels of eGFP:I-*Ppo*I-124-L expression in the testes from both Y-linked transgenic lines compared to wild type testes and to eGFP:I-*Ppo*I-124-L expression in the Ag(PMB)1-3 strains (Figure 2C and Supplementary Table S1). Consistent with the lack of X-shredder expression, we did not detect any significant sex bias, compared to the expected 50%, among progeny when transgenic males from each Y-linked strain were crossed to wild type G3 females (Y-pBac124L; χ^2^ = 12, *p* > 0.05, Y-AttP124L; χ^2^ = 12, *p* > 0.05; Figure 2A; Supplementary Table S2). These results highlight that, as would be expected from MSCI, the Y-chromosome is not permissive to transgene expression from the *beta2-tubulin* promoter during late spermatogenesis, similarly to the X-chromosome.

## Discussion

Genetic control strategies that aim to suppress wild populations of mosquito disease vectors have garnered significant interest, and field trials of a number of these systems, including classical SIT, IIT (*Wolbachia*-based sterility), and RIDL are now underway (Harris et al., 2012; Zheng et al., 2019). Synthetic sex-ratio distorters based on X-chromosome shredding have now been developed in *An. gambiae* (Galizi et al., 2014; Galizi et al., 2016) and more recently in *Drosophila melanogaster* and *Ceratitis capitata* (Fasulo et al., 2020; Meccariello et al., 2021). This system has been shown both theoretically and experimentally to be more efficient than classical SIT, in terms of the number of insects that need to be released (Schliekelman et al., 2005; Galizi et al., 2014; Burt and Deredec, 2018). In their most basic form, autosomal X-shredder constructs are self-limiting, and their release can potentially result in local and limited suppression if sufficient males are released over a long enough period. Nonetheless, field releases of fertile autosomal X-shredder males have not yet been conducted.

Here, we have evaluated the theoretical possibility whereby an autosomal X-shredder could convert into a self-sustaining, driving Y-chromosome after release. The requirements for such an event to occur include: 1) movement of the X-shredder to the Y-chromosome; 2) its subsequent expression from the Y during late stages of spermatogenesis at a level that result in X-chromosome shredding; and, 3) have a low enough fitness cost to male carriers such that the X-shredding effect is net beneficial to males carrying such a Y-chromosome. We reason that there are two possible mechanisms that could result in the linkage of the autosomal Ag(PMB)1 to the Y-chromosome: 1) a transposase-mediated remobilization of the transgene and 2) a large chromosomal rearrangement resulting in the reciprocal translocation between the region of the autosome containing the transgene and the Y-chromosome.

We have evaluated the potential of remobilization of the Ag(PMB)1 transgenic construct through transposition, mediated by the intact PB inverted terminal repeats (ITRs) on either side of the transgene cassettes, which were used for the initial generation of the strain. Their presence makes it at least theoretically possible that the Ag(PMB)1 transgene could remobilize from its autosomal position, if a source of the PB transposase occurs in *trans.* We therefore evaluated whether the PB transposase is present in the genomes of the laboratory Ag(PMB)1 and G3 colonies, and also in field-derived samples from Burkina Faso. We found no evidence of the complete PB transposase coding sequence in any of the samples we sequenced, suggesting that PB is not present at appreciable frequencies in *An. gambiae* mosquitoes sampled from nature. This result is supported by the long-term stability of the Ag(PMB)1 insertion site over 100 generations since its original construction, a result that suggests that other naturally occurring repetitive elements in the genome of the Ag(PMB)1 strain, including those that appear as PB-like by genome-wide translated nucleotide searches, are not functionally capable of PB transgene remobilization. Long-term stability of transgenic insertions in *An. gambiae* laboratory strains in the absence of experimentally provided PB transposase is well known, including through directed efforts of enhancer trapping through transgene mobilization (O'Brochta et al., 2011). The computational methods we developed to screen for the presence of PB transposase in genome sequencing data from wild type, transgenic and field samples could be adapted in the future for high-throughput screening of sequenced samples collected from the field to identify and quantify the presence of transgenic alleles without the need for fluorescence microscopy or complicated molecular genotyping protocols.

We were not able to test the second possibility of remobilization by chromosomal translocation, as these occur very rarely during meiosis. In over 7 years of both standard laboratory rearing (Galizi et al., 2014) and large-scale multi-generational cage studies of the Ag(PMB)1 strain (Facchinelli et al., 2019; Pollegioni et al., 2020), translocations involving the autosomal transgene and the Y-chromosome have never been detected–an event that would be noticeable in defined crosses of individuals since fluorescent transgenic individuals would only be male. This extends to scaled experimental conditions, in which large numbers of Ag(PMB)1 individuals were screened by high-throughput sorting of individuals using the COPAS sorter based on the 3xP3-DsRed marker and then subsequently separated by sex at the pupal stage (Burt and Deredec, 2018). This is, in part, expected because of how rarely such events occur, meaning that experimentally verified rates for such events are not readily available for the Ag(PMB)1 strain. When translocations between autosomes and the Y are desired, they can be artificially induced in the laboratory, for example with ionizing radiation, chemical agents, or UV radiation. This is commonly done for insects to link selectable phenotypes to the Y-chromosome, in so-called genetic sexing strains (GSSs). GSSs are developed so that males can be separated from females on a large scale in insect bio-factories that produce animals for genetic control programs, such as SIT (Gilles et al., 2014). This is done by linking selectable traits, for example insect color or high temperature tolerance, to the Y-chromosome using induced reciprocal translocations of mutant alleles located on autosomes. Once generated, these GSSs are then maintained in large numbers, with billions of insects being produced weekly. The large colony size makes it possible to detect rare events that result in the breakdown of linkage between maleness and selectable trait. One of two ways this can happen is through a “reverse” reciprocal translocation involving the previously modified Y-chromosome and an autosome (known as a type 2 recombination event) (Franz et al., 2005). Because such events lead to a breakdown of the genetic sexing system and restore male fertility of the semi-sterile males (arising from the translocation itself) and leading to their accumulation, their occurrence is tightly monitored in large-scale rearing operations. In the only report that quantified the rate of type-2 recombination and distinguished it from type-1 (which does not involve the translocated Y- chromosome and is more common among the two) in a GSS of the Mediterranean fruitfly *Ceratitis capitata*, the rate was estimated to be 10^−5^ or less, i.e., occurring in less than 1 out of 100,000 male individuals (Franz et al., 2005). In this case however, there are two significant factors that would indicate that the rate of an initial, uninduced autosome-Y translocation would be much lower. First, the rate of recombination in the GSS describes an event reversing a previously induced autosome-Y translocation, which is likely to be largely mediated by homologous sequences that are now present on the two translocated Y fragments; a homology that does not normally exist between autosomes and the Y chromosome. Therefore, the expected recombination rate resulting in a reciprocal translocation between an autosome and Y would be lower. Second, the reversion of the previously translocated autosomal fragment on the Y restores male fertility, that was first compromised by the translocation to the Y (because of gamete chromosomal imbalance–discussed below) (Franz et al., 2005). This means that type-2 recombinant males will have more viable offspring increasing their rate of occurrence in the population. Together, these factors suggests that the probability of a translocation event involving the autosomal Ag(PMB)1 transgene and the Y-chromosome in progeny of released males born in the field is expected to be much lower than 10^−5^. This rate would depend on the size of the Y-chromosome and the relative rate of recombination in the male germline, which in *An. gambiae* is approximately 1.6 cM Mb^−1^ for autosomes (Pombi et al., 2006) and similar between males and females (Benedict et al., 2003).

In the unlikely event that the transgene was to move to the Y-chromosome, we provide data regarding the expression of the X-shredder from this chromosome, and conclude that MSCI during spermatogenesis does affect the Y-chromosome of *An. gambiae*. Our results from two transgenic strains harboring the Ag(PMB)1 X-shredder transgene in two different positions on the Y-chromosome, reveal transcriptional suppression during late spermatogenesis from the *beta2-tubulin* promoter, complementing our previous work which confirmed this for the *An. gambiae* X-chromosome (Magnusson et al., 2011; Galizi et al., 2014; Papa et al., 2017). We found no evidence of X-shredder expression by quantitative RT-PCR, nor by fluorescence microscopy of transgenic testis. Offspring of transgenic males from both Y-linked strains therefore had sex-ratios similar to wild-type males. Hence, even if the Ag(PMB)1 transgene successfully moved to the Y-chromosome by transposition (first requirement for a driving Y) it is unlikely that the X-shedder would be active. Since MSCI-factors regulating transcriptional suppression physically spread across the sex chromosomes after becoming localized on their unsynapsed axes (Ichijima et al., 2011; Ichijima et al., 2012), it is also expected that translocated autosomal fragments would become suppressed by MSCI during meiotic stages of spermatogenesis. Therefore, the weight of evidence argues strongly against the likelihood of movement of the Ag(PMB)1 transgene to the Y chromosome, particularly via transposition. However, the equally necessary prerequisite for a pathway to a driving Y, namely expression of the X-shredder on the Y chromosome during male meiosis, seems highly implausible based on the evidence presented here.

The final requirement for a Y-linked X-shredder to spread through populations is that its movement to the Y-chromosome and subsequent expression from it would have no significant fitness costs to males harboring it. Such fitness costs would counteract the theoretical advantage gained by the Y-linked X-shredder from increased transmission through elimination of X-bearing sperm. Among the factors determining these fitness costs, the largest contributors would likely be the mechanism leading to Y-linkage and the outcomes of this movement on each chromosome. Reciprocal translocations between an autosome and the Y-chromosome have be found to result in significant male fertility costs (Roukos and Misteli, 2014). Because of the simultaneous segregation of non-homologous centromeres (adjacent-1 segregation) during meiosis, only 50% of the offspring produced by males are genetically balanced, i.e., males are 50% sterile (Yamada et al., 2012). In certain cases, this semi-sterility can be even higher, for example in an *An. arabiensis* GSS showing 73.3% male sterility (Yamada et al., 2012). Therefore, a Y-linked X-shredder that arose through a translocation event would likely display sufficiently high male fertility costs that it would rapidly disappear from the population. For transposition-mediated Y-linkage male fitness costs cannot be predicted *a priori*, as gamete balance and genic content would depend on both the excision event (i.e., how much of the surrounding chromosome is excised) and on the integration position on the Y-chromosome (i.e., subsequent knock-out of genes essential for male fitness such as the male-determining gene).

In summary, the findings of the current study support the low probability of transgene remobilization from the autosome to the Y-chromosome. Moreover, even if such a rare event occurred, where the X-shredder would become linked to Y-chromosome, activity of the X-shredder at the required stage of spermatogenesis would likely be impeded via chromosome wide suppression of gene expression on meiotic sex chromosomes. Our results also show that prospects for the successful building of self-sustaining Y-linked X-shredders for mosquito control in the future will need to find ways to circumvent this transcriptional suppression, for example using alternative germline specific promoters (Taxiarchi et al., 2019). Finally, more studies and methods are needed to systematically explore how population dynamics of released elements could be impacted by spontaneous genomic changes, such as transgene remobilization, done in a way that is technology-specific and relevant.

## Methods

### Mosquito Rearing

Wild-type *An. gambiae* strain (G3) and transgenic mosquito strains were reared under standard conditions at 28°C and 80% relative humidity with access to fish food as larvae and 5% (wt/vol) glucose solution as adults. For egg production, young adult mosquitoes (3–5 days after emergence) were allowed to mate for at least 6 days and then fed on mice. Three days later, an egg bowl containing rearing water (dH_2_O supplemented with 0.1% pure salt) was placed in the cage. One to 2 days after hatching, the larvae (L1 stage) were placed into rearing water containing trays. All animal work was conducted according to UK Home Office Regulations and approved under Home Office License PPL 70/8914.

### Assaying Transgene Stability

PCRs were performed on selected transgenic and non-transgenic siblings that were screened twice during larval development for the DsRed phenotype. DsRed-positive and -negative individuals were examined by duplex and simplex PCR (GoTaq DNA polymerase, Promega). These PCR reactions amplified: 1) a fragment consisting of the wild-type genomic insertion site of the transgene i.e., the empty site that occurs in all individuals, regardless of whether they are transgenic or not, as a positive control; 2) a fragment of the internal DsRed marker; 3) fragments consisting of the known downstream (or upstream) flanking regions of the transgene (Supplementary Figures S3, S4). DNA was purified using the Qiagen Blood and Tissue kit.

### Mosquito Whole Genome Sequencing and Read Mapping

*Anopheles gambiae* WGS reads from 81 individuals collected in Burkina Faso in 2012 were downloaded from the European Nucleotide Archive (Accession: PRJEB1670; Supplementary Table S3). WGS data from the G3 laboratory colony were downloaded from the SRA (Accession: PRJNA397539). Genomic DNA from 10 Ag(PMB)1 individuals was extracted using the Blood and Tissue Kit (Qiagen). For each sample, 100 ng of input gDNA was sheared using Covaris for a 350 bp insert size. Library preparation was performed using the Illumina TruSeq Nano kit. Each sample was tagged with a unique barcode, followed by three 2 × 150 bp High Output V2.5 paired-end sequencing runs on the Illumina NextSeq550 platform (PoloGGB, Sienna, Italy), obtaining an average of 265M reads per sample. WGS data from the Ag(PMB)1 have been deposited at NCBI SRA (Accession: PRJNA594202). Fastq reads were quality checked with FastQC (Andrews, 2015) and converted to fasta format. Reads were then mapped against the *vasa* driven piggyBac plasmid (Volohonsky et al., 2015) using blast blast-2.2.26/bin/blastall -i db. fa -d sample. fasta -p blastn -F ″m L″ -U T -e 1-e4 -a 40 -v 5 -b 40000 -K 40000. Only alignments with 98% identity over the entire read length were kept. Coverage was computed for each sample and normalized to the read depth of the most deeply sequenced sample using the following formula X_i_ = X_i_/(X_i_/X_max_). To clarify plotting, read depth is reported every 10 bp.

### Generation of Y-Chromosome Linked X-Shredder Transgenic Strains

The YpBac-β2-^gfp^124L transgenic strain was generated as described in Galizi et al. (2014). Briefly, *An. gambiae* G3 embryos were injected with a mixture of 0.2 μg/μl of the pBac(3xP3-DsRed)β2-eGFP:I-*Ppo*I-124L plasmid and 0.4 μg/μl of helper plasmid containing a *vasa*-driven piggyBac transposase (Volohonsky et al., 2015). The hatched larvae were screened for transient expression of the DsRed marker and positives (∼54%) crossed to wild-type mosquitoes. F1 progeny were analyzed for DsRed fluorescence and positives were crossed individually with wild-type mosquitoes to obtain transgenic lines. The transgene of one strain derived from a G0 male was identified that was transmitted exclusively to F1 sons, indicating Y-chromosome integration. The stain, now called YpBac-β2-^gfp^124L was established and maintained by crossing to wild type females. The YattP- β2-^gfp^124L strain was generated by co-injecting the pBac(3xP3-DsRed)β2-eGFP:I-*Ppo*I-124L construct with a *vasa*2-driven ΦC31 integrase helper plasmid (Volohonsky et al., 2015) into eggs of a strain containing a Y-chromosome AttP docking site (Bernardini et al., 2014). Crosses and screening were performed as above.

### Sex Ratio and Fertility Assays

To assay adult sex ratio, transgenic males of each line were crossed to wild-type females. In all crosses, mosquitoes were allowed to mate for 3–5 days after the blood meal and gravid females were placed individually in oviposition cups. Larvae were reared to adulthood and sex was counted. The number of eggs laid as well as the number of larvae hatching were also counted, but only for the YattP-β2-^gfp^124L to assay male fertility. The difference in sex bias among progeny of the Y-linked strains was tested independently to the expected 50% male ratio, using the chi-square test.

### qRT-PCR Analysis

qRT-PCRs were performed on mosquito total RNA as described in Galizi et al. (2014). Briefly, 10 pairs of testes from each transgenic strain were pooled to constitute a biological replicate for total RNA and protein extraction using TRI reagent (Ambion). RNA was reverse-transcribed using Superscript II (Invitrogen) after TURBO DNA-free (Ambion) treatment following the manufacturer’s instructions. Quantitative real-time–PCRs (qRT–PCR) analyses were performed on cDNA using the Fast SYBR-Green master mix on a StepOnePlus system (Applied Biosystems). Ribosomal protein Rpl19 gene was used for normalization. At least two independent biological replicates from independent crosses were subjected to duplicate technical assays. We used primers RPL19Fwd (5′-CCA​ACT​CGC​GAC​AAA​ACA​TTC-3′), RPL19Rev (5′-ACC​GGC​TTC​TTG​ATG​ATC​AGA-3′), eGFP-F (5′-CGG​CGT​GCA​GTG​CTT​CA-3′), and eGFP-R (5′-CGG​CGC​GGG​TCT​TGT-3′). Internal normalization was done as in (Galizi et al., 2014) to the RPL19 ribosomal genes and normalized to expression from wild type testis.

## Data Availability

The datasets presented in this study can be found in online repositories. The accession numbers are: SRA, PRJNA594202, DQ236240.1, PRJEB1670, PRJNA397539. Other datasets presented in this study can be found in online repositories. The names of the repository/repositories and accession number(s) can be found in the article/Supplementary Material.
