# Supplementary figures and images for: The Potential for a Released Autosomal X-Shredder Becoming a Driving-Y Chromosome and Invasively Suppressing Wild Populations of Malaria Mosquitoes
Source: Front Bioeng Biotechnol. 2021 Dec 3;9:752253. doi: 10.3389/fbioe.2021.752253 (PMC8698249; doi:10.3389/fbioe.2021.752253)

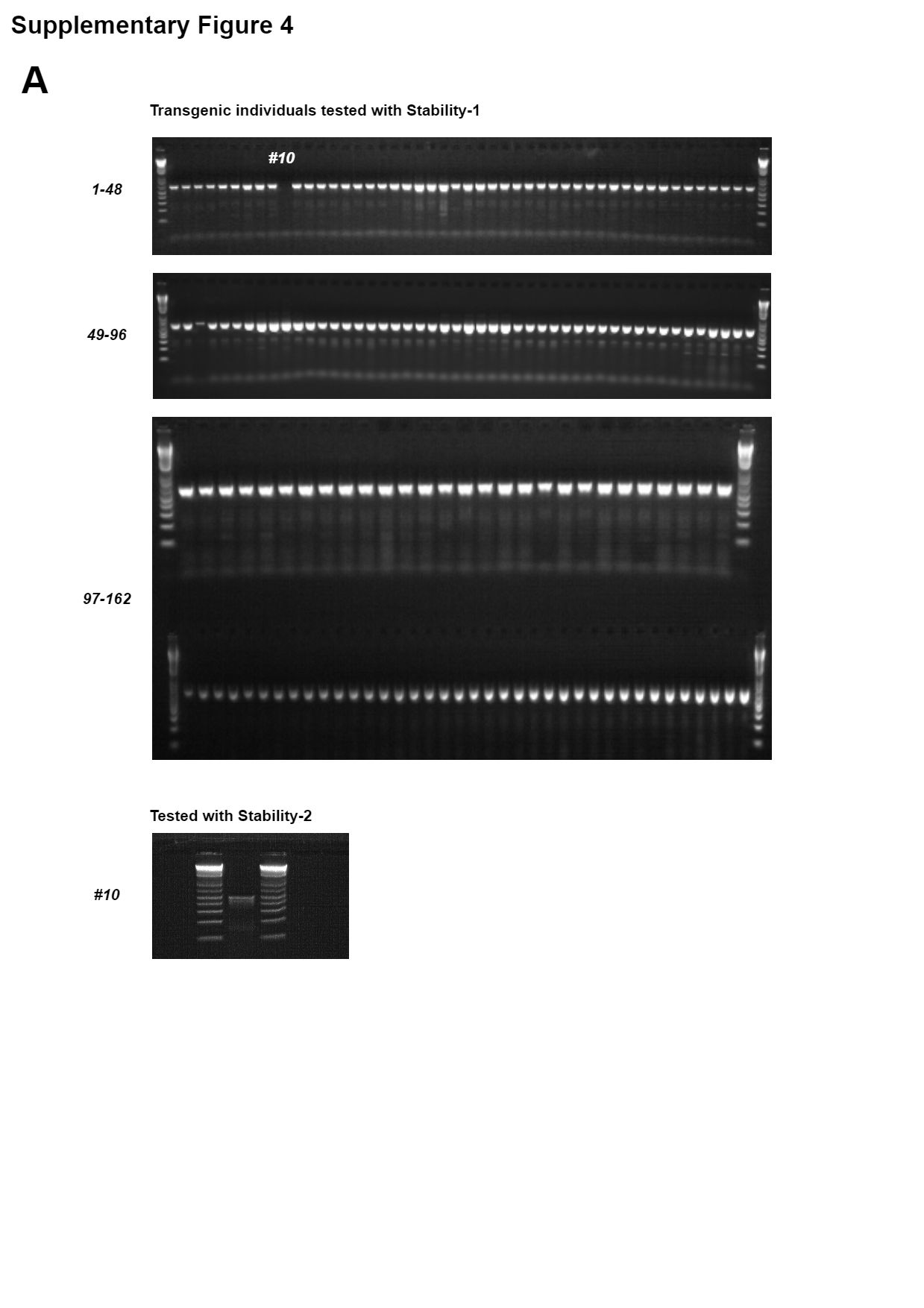

Supplement: Supplementary file 2 [file Image3.jpg]

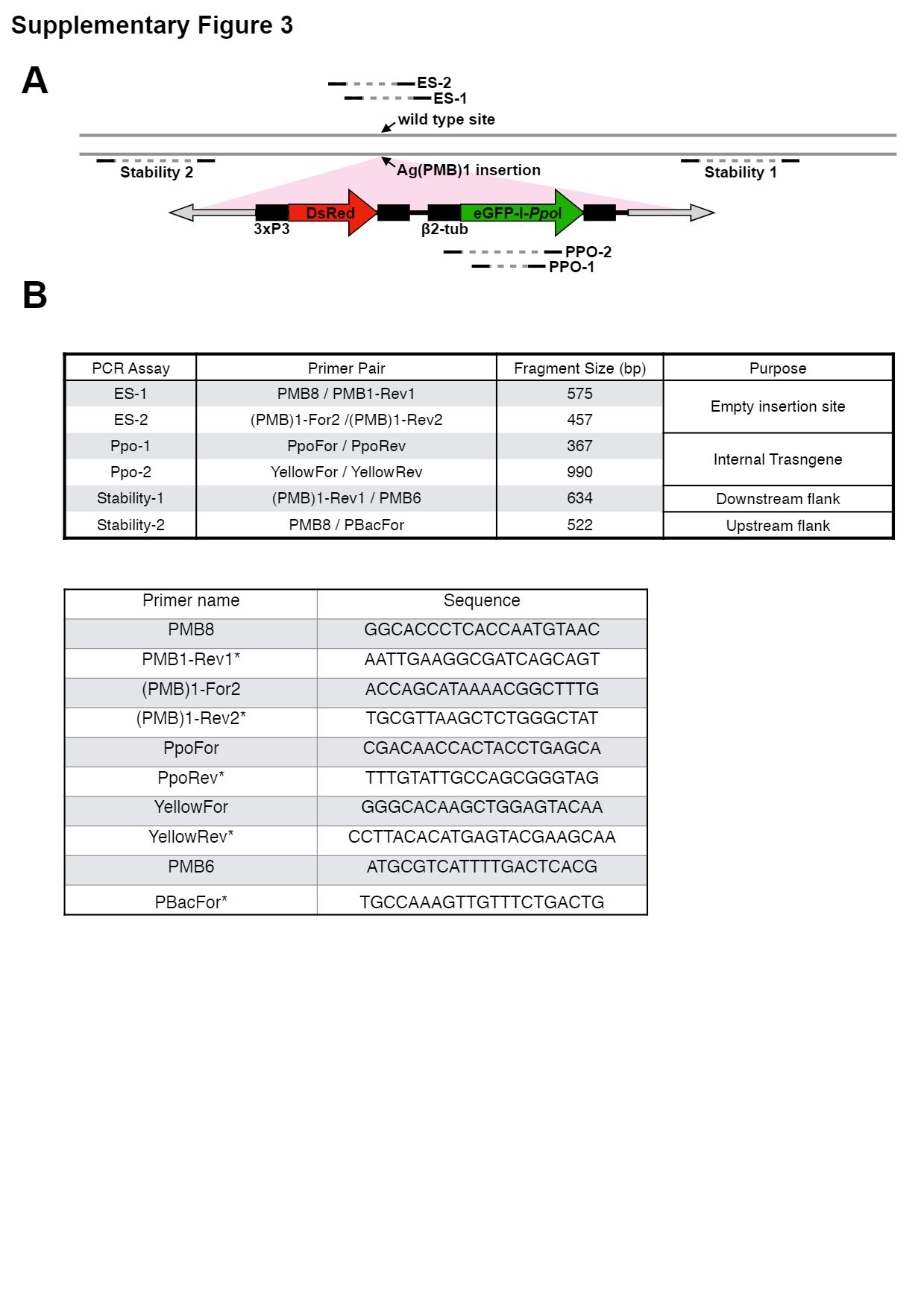

Supplement: Supplementary file 3 [file Image2.jpg]

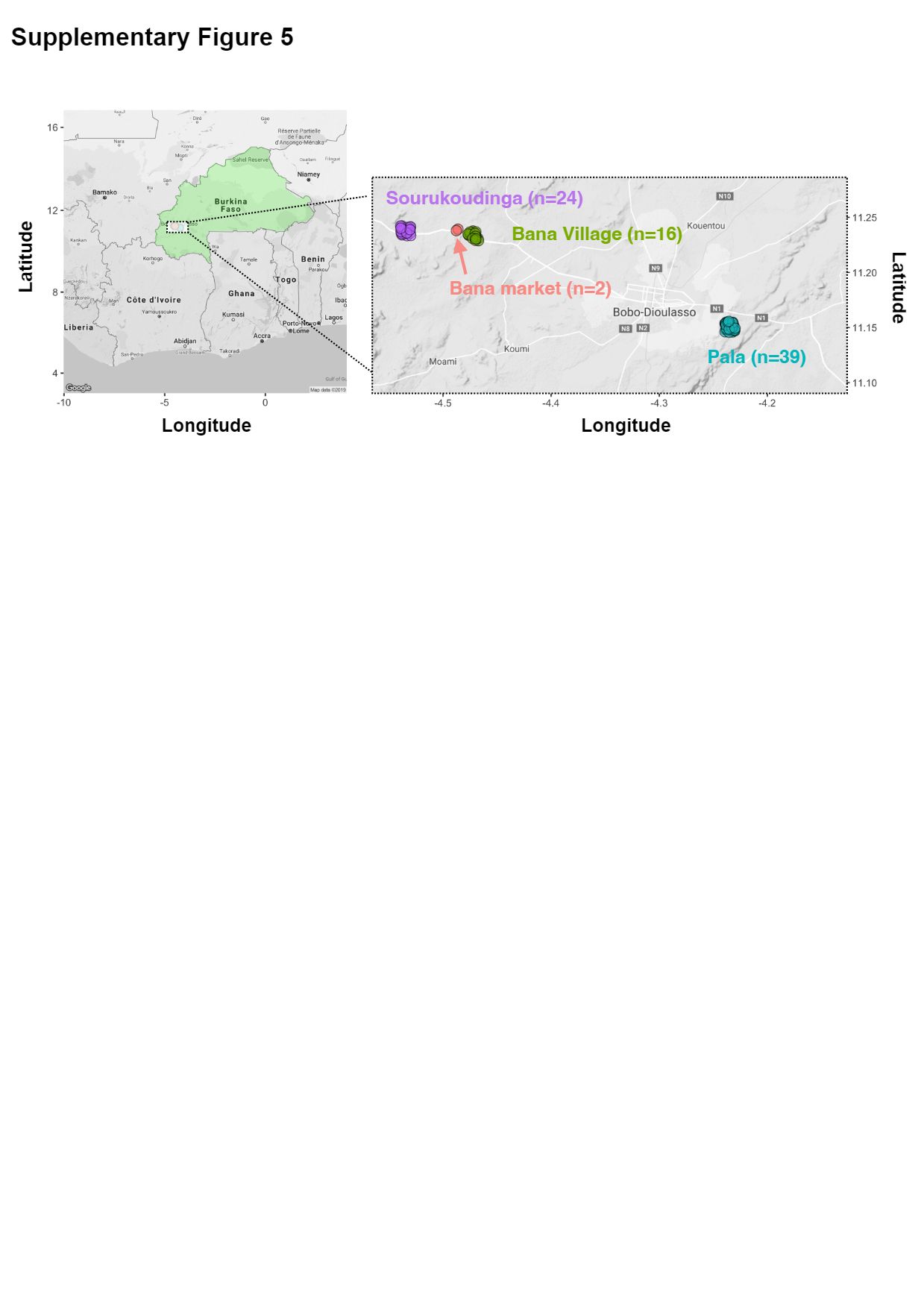

Supplement: Supplementary file 5 [file Image4.jpg]

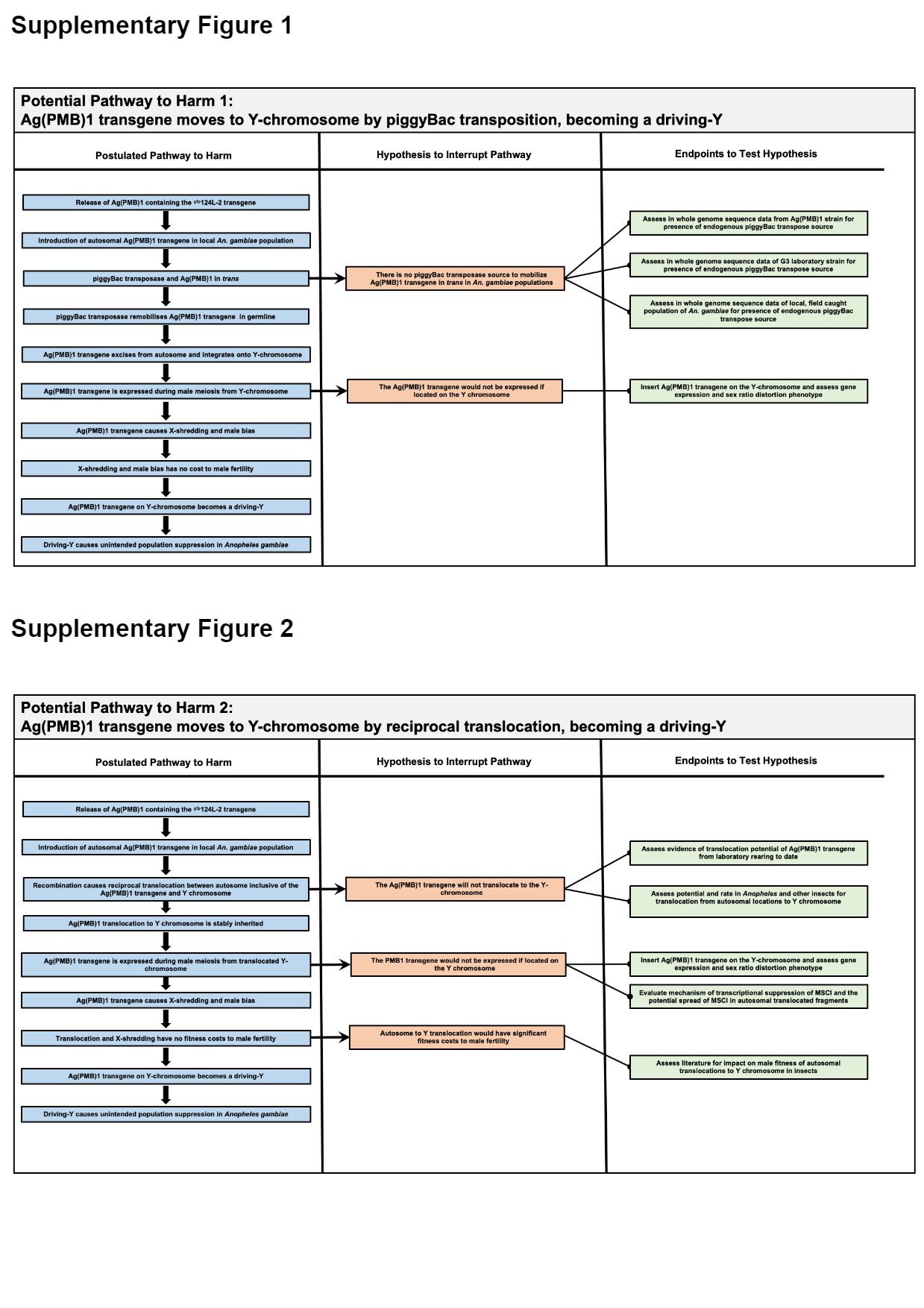

Supplement: Supplementary file 6 [file Image1.jpg]
